# Fluorescent Lactic Acid Bacteria and Bifidobacteria as Vehicles of DNA Microbial Biosensors

**DOI:** 10.3390/ijms18081728

**Published:** 2017-08-08

**Authors:** José María Landete, Juan Luis Arqués

**Affiliations:** Dpto. de Tecnología de Alimentos, Instituto Nacional de Investigación y Tecnología Agraria y Alimentaria (INIA), 28040 Madrid, Spain; landete.josem@inia.es

**Keywords:** DNA microbial biosensors, lactic acid bacteria, bifidobacteria, fluorescent proteins, cloning vectors, clustered regularly interspaced short palindromic repeats

## Abstract

Control and quantification of effector molecules such as heavy metals, toxins or other target molecules is of great biotechnological, social and economic interest. Microorganisms have regulatory proteins that recognize and modify the gene expression in the presence or absence of these compounds (effector molecules) by means of binding to gene sequences. The association of these recognizing gene sequences to reporter genes will allow the detection of effector molecules of interest with high sensitivity. Once investigators have these two elements—recognizing gene sequences and reporter genes that emit signals—we need a suitable vehicle to introduce both elements. Here, we suggest lactic acid bacteria (LAB) and bifidobacteria as promising carrier microorganisms for these molecular biosensors. The use of fluorescent proteins as well as food-grade vectors and clustered regularly interspaced short palindromic repeats (CRISPR) are indispensable tools for introducing biosensors into these microorganisms. The use of these LAB and bifidobacteria would be of special interest for studying the intestinal environment or other complex ecosystems. The great variety of species adapted to many environments, as well as the possibility of applying several protocols for their transformation with recognizing gene sequences and reporter genes are considerable advantages. Finally, an effort must be made to find recognizable gene sequences.

## 1. Introduction

Biosensors, sometimes also referred to as bioreporters or genosensors, are microorganisms, cell cultures or cell lines, often genetically engineered, with activity that reflects changes in environmental conditions in a dose-dependent manner [1].

**Figure 1:**
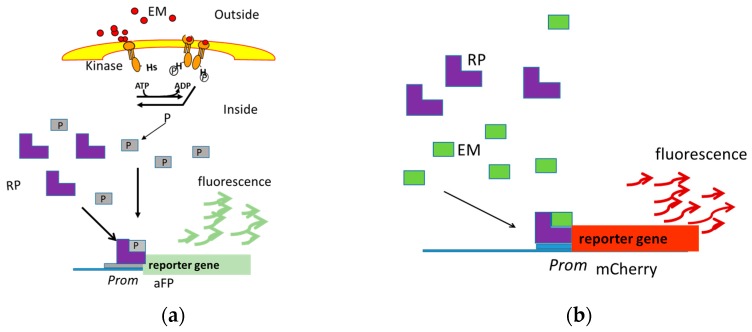
The reporter genes *aFP* and *mCherry* are transcribed when the effector molecule present in the cell’s environment is recognized by a transcriptional regulator that is capable of binding to DNA, modifying the transcription. Two-Component Systems (**a**) and Two-Domain Proteins (**b**) are systems implicated in effector molecule recognition. EM, Effector Molecule; P, Phosphate; RP, Receptor Protein; *Prom*, Promotor; *aFP*, Anaerobic Fluorescent Protein.

The origin of biosensors can be found in the adaptive responses of living organisms, which are mediated by transcriptional regulators that recognize effector molecules binding to the DNA modifying the transcription [2]. The promoter gene in a normal bacterial cell is linked to other genes that are then likewise transcribed, and then translated into proteins that help the cell in either combating or adapting to the agent to which it has been exposed [3]. They contain two essential genetic elements; a promoter sequence (biosensor) and a reporter gene (bioreporter). The reporter gene is turned on (transcribed) when the target agent or effector molecule present in the cell’s environment is recognized by a protein (transcriptional regulator) that is capable of binding to DNA, modifying the transcription [4] (Figure 1). Biosensors that employ nucleic acid interactions can be termed genosensors. However, we prefer the term “molecular biosensors”, which would be defined as DNA-based sensors in which live microorganisms are used as vehicles, and where the presence of a recognizing protein that binds DNA and regulate transcription is also necessary.

The use of molecular biosensors represents a great opportunity to detect and even quantify molecules of interest due to their biotechnological implications, or their toxicity, with great sensitivity [5,6]. Here, we propose intensifying research on the use of fluorescent lactic acid bacteria (LAB) and bifidobacteria as biosensor vehicles through the use of genetic engineering. Taking advantage of their enzymatic activities to detect metabolites such as organic acids and sugars [7], LAB have been used as biosensors, although the metabolites detected are very limited, and their sensitivity is low. The use of genetic engineering in the development of these sensors would allow to detect many more metabolites and with much more sensitivity, being able to transfer these biosensors to LAB and bifidobacteria. In addition, the use of molecular biosensors would allow the potential detection of any metabolite.

## 2. Reporter Genes Used in Lactic Acid Bacteria and Bifidobacteria

In the case of a bioreporter, genes have been removed and replaced with a reporter gene. Reporter genes are used as an indication of whether a certain gene has been taken up by, or expressed in, the cell or organism population. The generated signal indicates that the bioreporter has sensed a particular effector molecule in its environment, and this signal is proportional to the concentration of the unique chemical or physical agent to which it has been exposed.

Although colorimetric reporters and luciferin–luciferase light-emitting systems have been used for real-time imaging of bacteria [8,9], including LAB and *Bifidobacterium* strains [10,11], fluorescent reporter systems have several advantages since do not require any substrate or additional cofactors for fluorescence, and are considered more versatile as genetically encoded probes [12]. They have been isolated and manipulated for several applications, developing different fluorescent proteins from several sources which result in a wider range of colors, increasing the spectrum of possibilities of these proteins as molecular probes [13,14,15,16,17,18]. A vast range of fluorescent proteins that feature fluorescence emission spectral profiles spreading from blue to red have been developed during the last decades [19,20,21]. Different factors must be studied in order to choose the best fluorescent variant to use for a given assay and genera of bacteria, including the brightness, protein stability, pH and temperature stability, as well as the potential interference of the fluorescent protein on the molecule studied [22]. Oxygen-independent flavin mononucleotide-based fluorescent proteins [23,24] are promising probes, which would be suitable for application in a broad range of bacteria, including anaerobic bacteria [25,26,27].

The use of fluorescent proteins as a visible marker and as a transcriptional reporter to monitor bacterial gene expression in real-time in LAB and *Bifidobacterium* spp. in living cells has been addressed through different strategies [26,27,28,29,30,31,32,33,34,35,36,37].

## 3. Identification of Biosensors

### 3.1. LAB and Bifidobacteria as Vehicles for Molecular Biosensors

LAB and bifidobacteria are involved in the manufacture of fermented foods from agricultural raw materials such as milk, meat, vegetables and cereals. Both are ubiquitous inhabitants of the gastrointestinal tract, vagina and mouth of mammals, including humans, and are the most common microbes used as probiotics [38,39,40,41].

The main advantages of the use of LAB and bifidobacteria for their use as biosensor vehicles are: (1) they can be genetically manipulated: the use of food-grade vectors and clustered regularly interspaced short palindromic repeats (CRISPR) would be the main options [42,43]; (2) strains of *Lactobacillus* and *Bifidobacterium* are the most common probiotics used in food products [44]; (3) the great diversity of species of LAB and bifidobacteria allow their use in many different habitats [45]; (4) these microorganisms are widely known both physiologically and genomically, with many genomes of LAB and bifidobacteria sequenced; (5) they have a good image and are not usually rejected; and (6) many of them are listed as generally recognized as safe (GRAS) by Food and Drug Administration (FDA), or as qualified presumption of safety (QPS) by the European Food Safety Authority (EFSA), allowing their use in foods and intestinal environmentals [46].

**Figure 2:**
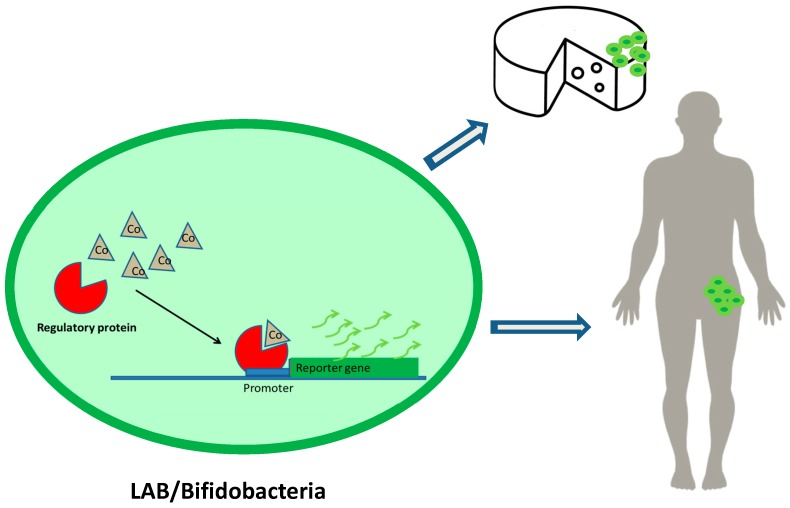
Detection of cobalt in food and living beings through the use of lactic acid bacteria (LAB) or bifidobacteria as DNA microbial biosensor vehicles.

Figure 2 shows an example of the use of LAB or bifidobacteria as DNA microbial biosensor vehicles. Regulatory proteins recognize the effector molecule (Cobalt), joining the promoter sequence and inducing expression of the reporter gene. The development of reporter genes from DNA sequences of LAB and bifidobacteria together with food-grade vectors and/or CRISPR systems would allow their application in food and humans.

### 3.2. Genetic Engineering for Molecular Biosensors

**Figure 3:**
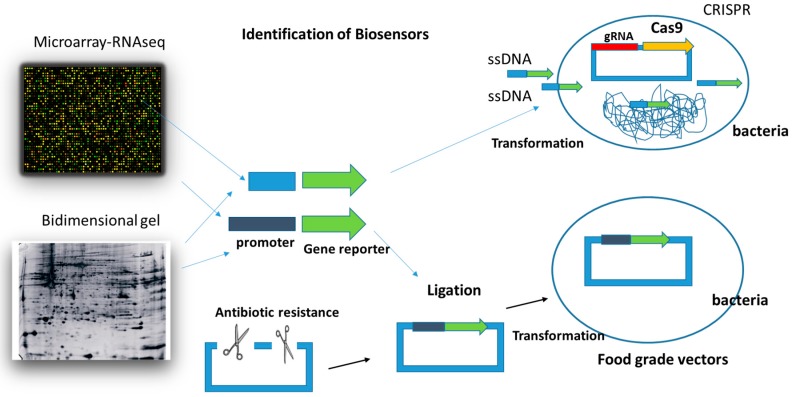
Transcriptomic techniques (Microarrays or RNAseq) and proteomic techniques are the main approaches to identify promoter sequences that in the presence of specific effector molecules are involved in the increase or decrease of the gene expression. The use of food-grade vectors and the CRISPR methodology for the genetic manipulation of LAB and bifidobacteria is suggested in the present work in order to use them as biosensor vehicles in foods and living beings.

Advances in gene technology allow their modification by introducing new genes, or modifying their metabolic functions. These changes can lead to improvements in food, technology and health. Traditionally, antibiotic resistance genes have been used as markers for the selection of vectors in research laboratories. However, for legal and ethical reasons, transfer of genes conferring resistance to antibiotics is not acceptable for food or clinical applications, and alternatives must be sought [43,47]. The use of food-grade vectors and the CRISPR methodology (Figure 3) for the genetic manipulation of LAB and bifidobacteria in order to use them as biosensor vehicles in foods and living beings is suggested in the present work.

#### 3.2.1. Food-Grade Cloning Vectors

Food-grade vectors are vectors with DNA from GRAS organisms and require the use of selective markers that allow selection and maintenance in the host [47]. Moreover, these vectors must be devoid of any antibiotic resistance marker that could compromise their applications in food [43]. Consequently, the vectors should contain selection markers that are acceptable in the food industry; these are described as food grade. These markers can be selected because they confer a new phenotype, or because they restore impaired functions [48,49]. Several food quality systems have been proposed in LAB. The first resistance markers proposed were immunity markers to bacteriocin production. These are dominant markers, as in the case of markers for resistance to nisin [50] or lactacin F [51].

Food-grade vectors have been developed to meet industrial demands for GRAS recombinant products. Although any genetic manipulation of an organism creates a geneticaly modified organism (GMO), food-grade modifications employ its own DNA or DNA from GRAS organisms, might not be as ill perceived as a non-food-grade genetic modification [43,47]. Self-cloning, i.e., the re-introduction of DNA from a host that has been modified, or is closely related to the same species strain, was excluded from the European Union Directive on the contained use of genetically modified microorganisms (CCA-219, 1990) [48]. Moreover, these microorganisms have been recognized as GRAS/QPS microorganisms. Organisms that have been modified by self-cloning are not considered to be GMOs, but are considered safe and suitable for food applications.

#### 3.2.2. Clustered Regularly Interspaced Short Palindromic Repeats

The prokaryotic CRISPR-Cas9 can be regarded as an immune system for bacteria and archaea, as it efficiently cleaves foreign DNA entering the cell, such as phage or plasmids [52,53]. CRISPR-Cas9 has been shown to mediate efficient genome editing in a wide variety of organisms [54,55].

The CRISPR-array is transcribed and processed yielding RNA fragments, called CRISPR-RNA (crRNA). The crRNA serves to direct the Cas nuclease to the target site, and the presence of a specific protospacer-adjacent motif (PAM) results in Cas9-mediated cleavage of the target sequence. In type-II CRISPR–Cas systems, Cas9 will form a dual-RNA complex as Cas9 complexes with crRNA and a trans-activating CRISPR RNA (tracrRNA), which is required for Cas9 nuclease activity. The crRNA can be homed to user-defined locations in the genome to promote double-stranded breaks to eliminate unedited DNA [42]. The development and optimization of CRISPR–Cas9 selection in *Lactobacillus reuteri* ATCC PTA 6475 has been reported [56]. DNA editing could be generated in the chromosome of LAB by single-stranded DNA (ssDNA) recombineering [56,57]. ssDNA recombineering requires inducible expression of a phage-derived ssDNA-binding protein (RecT or β). Once the oligonucleotide is in the cell, the ssDNA-binding protein protects the oligonucleotide from degradation by host nucleases and aids in forming a complex between the oligonucleotide and the lagging strand template DNA. The co-transformation of a recombineering oligonucleotide and a CRISPR-target plasmid, a single-step approach, will yield recombinants when ssDNA recombineering efficiencies are optimal [42,56,57,58]. Thus, the power of these systems to perform highly efficient alterations targeted at genome sequences could be used in the development of molecular biosensors.

### 3.3. Microarray-RNAseq and Bidimensional Gel to Develop Molecular Biosensors

A very interesting aspect concerning the creation of biosensors is the identification of gene sequences or promoters that, in the presence of specific effector molecules, are involved in the increase or decrease of the gene expression. For example, the identification of a promoter that in the presence of copper induces the expression of a gene will allow the use of that promoter, together with a reporter gene, for the identification and quantification of the presence of cobalt [59]. In order to detect the promoters or gene sequences that induce expression when it cannot be found in the literature, the use of transcriptomic techniques (Microarrays or RNAseq) [60] and proteomic techniques (two-dimensional gels) [61,62] (Figure 3) are fundamental approaches. In the case of microarrays or RNAseq, the gene expression is analyzed in the bacteria that we want to use as a vehicle in the presence and absence of the molecule that we want to detect. Thus, we will grow the vehicle bacterium in the presence and absence of cobalt (for example), and the genes that are induced at a certain level will be susceptible promoters to be used as promoters of reporter genes in the biosensors for the identification of cobalt. Moreover, in an ideal scenario, they would only be expressed in the presence of the molecule we are looking for, in this case, cobalt. The other option is to use the promoters of the proteins that we detected in the two-dimensional gels in the presence of the molecule of interest (cobalt), which we did not detect in the absence of this molecule.

There are various problems for the selection of these sequences. Therefore, it is most advisable to look for promoters that are induced in the same bacterial strain that is to be used as a vehicle, although strains of the same species or even related bacteria could work. However, the bacteria need a sensory protein that recognizes that molecule and a regulatory protein that binds to DNA by inducing or repressing transcription. Thus, we need sensory and regulatory proteins, usually two-component systems, which recognize the molecule of interest, as previously proposed [43]. At other times, they are proteins of two domains able to detect the molecule and modify the transcription.

It may happen that a promoter that is good as a sensor in a particular bacterial strain cannot function in another strain of the same species, or in another species, and this will surely occur in phylogenetically distant bacteria. The carrier bacterium would not have the molecular machinery that allows the detection of the target molecule (hence the term molecular biosensor). In this case, we can introduce in a vector the two-component system or the gene of the protein of two domains involved in the recognition of the molecule of interest and the increase in the transcription (Figure 1). For example, the introduction of the *NisK*/*NisR* genes allows the induction of genes of interest by nisin with the nisin promoter [43]. Another option is to look for new promoters induced in this bacterium in their presence.

With the results obtained by means of Microarray-RNAseq and bidimensional gel, regulatory proteins could be identified analyzing the genome of the bacteria and gene knockout or complementation of the potential genes.

The development of vectors that, besides the promoters, have the molecular machinery (a sensory protein that recognizes that molecule and a regulatory protein that binds to DNA by inducing or repressing transcription) for the detection of the molecule and activation of the expression would be of great interest.

## 4. LAB and Bifidobacteria as Biosensors

Although there are examples of molecular biosensors of LAB in the literature, these are scarce. They are usually biosensors of heavy metals and bacteriocins (Table 1). One cadmium-induced gene (*csrA)* was detected in *Enterococcus faecalis* for pollutant detection. The *crsA* mRNA was barely present in unstressed *E. faecalis* cells grown in M17-glucose medium, but accumulated at higher levels in cadmium-treated cells. Mercury also had an effect on *csrA* expression, whereas lead, copper and manganese induced *csrA* expression only at the highest doses tested. The results shown by Laplace et al. [6] suggest that biosensors may have potential applications for environmental monitoring. Similarly, copper homeostasis is controlled by the *cop* operon in *Enterococcus hirae* [59]. Induction of the *cop* operon was also assessed in vivo with a biosensor containing a *lux* reporter system under the control of the *E. hirae cop* promoter. Half-maximal induction of this biosensor was observed at 5 µM media copper, which delineates the ambient copper concentration to which the *cop* operon responds in vivo. However, these authors detected genes that are induced by analysis of the transcription. Two-dimensional gel electrophoresis has been employed to detect changes in the proteome in response to copper in order to identify components of the copper homeostatic mechanism of *Lactococcus lactis* [63]. Three proteins up-regulated by copper were identified: glyoxylase I (YaiA), a nitroreductase (YtjD), and lactate oxidase (LctO). The promoter regions of these genes feature *cop* boxes of consensus TACAnnTGTA, which are the binding site of CopY-type copper-responsive repressors. They can then be used to detect copper using these promoters and a gene reporter.

The other outstanding feature in the use of biosensors in LAB are bacteriocins. A method for determining ultralow amounts of nisin in food samples has been developed [5]. Modified bacterial luciferase operon *luxABCDE* was placed under control of the nisin-inducible *nisA* promoter in plasmid pNZ8048, and the construct was transformed into the *L. lactis* strains NZ9800 and NZ9000. The *nisRK* genes of these strains allow them to sense nisin and relay the signal to initiate transcription from the *nisA* promoter. The resulting luminescence can be directly measured from living bacteria without the addition of exogenous substrates. The sensitivity of the nisin bioassay was 0.1 pg/mL in pure solution and 3 pg/mL in milk. Nisin-producing bacteria were also detected [64]. Fluorescence-activated cell sorting was used to isolate mutants of *L. lactis* LAC275. This strain harboured the GFP encoding gene under the *nisA* promoter and the nisin signal transduction *nisRK* genes and the nisin concentration can be correlated to GFP fluorescence [65].

Other molecular biosensors can be used for other purposes, such as studying the gene expression of transiting bacteria in human fecal specimens. Promoter expression has been monitored during cell growth, and the variable luciferase activities detected, demonstrating how certain genes are expressed in the gastrointestinal tract [66]. As an example of the potential of the technique, biosensors could be used to measure antibodies, enzymes, tumor necrosis factor or proteins of interest [67,68,69,70].

A close correlation between agmatine concentration and fluorescence was observed when GFP was used as reporter in the *E. faecalis aguR*/P*_aguB_* controlled expression system. Then, the induction of agmatine in *E. faecalis* could be used for the overexpression of recombinant proteins [71].

Finally, Guglielmetti et al. [72] constructed a bifidobacterial biosensor that could be used to analyze the metabolic state of cells. That bioluminescent *Bifidobacterium longum* is a tool for studying the physiological state of anaerobic bacterial cells under different environmental conditions.

## 5. Conclusions and Perspectives

LAB and bifidobacteria can be used as biosensor vehicles for the detection of effector molecules, providing information about the improvement or worsening of some functional foods or living organisms. Through the use of food-grade vectors and the CRISPR system, we can successfully introduce the biosensors into LAB and bifidobacteria with great sensitivity. Transcriptomics and proteomics help us to develop these vectors by finding the gene sequences and proteins that recognize the target molecules detected by biosensors.

The use of molecular biosensors in LAB and bifidobacteria will provide a cost-effective, quantitative method for rapid and selective detection and monitoring of chemical and biological agents in applications as far-ranging as fermentation, environmental monitoring, food safety, precision agriculture, and process monitoring and control. Their attractiveness lies in the fact that they can often be implemented in real-time on-line bioassays within intact, living cell systems, thus providing a unique and revolutionary new perspective on bacterial and mammalian physiology and intracellular interactions.

Although there are already works on the use of LAB as biosensor vehicles, these works are scarce, and are limited to the detection of heavy metals and bacteriocins. Hence, biotechnology laboratories with expertise in LAB and bifidobacteria need to focus on the development of new vectors that will allow to control fermentation or the presence of toxic molecules, for example in the gastrointestinal tract.

Advances in the sequencing of the genomes of LAB and bifidobacteria, and in transcriptomic and proteomic techniques on these microorganisms, greatly facilitate the development of biosensors in these microbial groups. However, new and renewed efforts should be made for the development of food-grade vectors, and fundamentally in the development of the emerging CRISPR technique.

## Figures and Tables

**Table 1:** Some examples of molecular biosensors in LAB and bifidobacteria.

Bacteria	Reporters	Promoters	Effector	Reference
*Lactococcus lactis* NZ9800/NZ9000	lux reporter system	*Pnis*	Nisin in food samples	[5]
*Enterococcus faecalis* JH2-2	^32^P-labeled probe of csrA cDNA	*csrA*	Heavy metals	[6]
*Enterococcus hirae*	lux reporter system	*cop*	Copper	[59]
*Lactococcus lactis* IL1403	-	*YaiA, YtjD,* LctO	Copper	[63]
*Lactococcus lactis* NZ9800	lux reporter system	*Pnis*	Nisin producers	[64]
*Lactococcus lactis* LAC275	GFP	*Pnis*	Nisin	[65]
*Lactobacillus casei* DN-114 001	lux reporter system	*ccpA, dlt, ldh, lacT*	Changes in the gastrointestinal tract	[66]
*Enterococcus faecalis*	GFP	*aguB*	Agmantine	[68]
*Bifidobacterium longum*	lux reporter system	phage T5 promoter	Carbohydrates	[69]
